# Mapping spontaneous facial expression in people with Parkinson’s disease: A multiple case study design

**DOI:** 10.1080/23311908.2017.1376425

**Published:** 2017-09-08

**Authors:** Sarah D. Gunnery, Elena N. Naumova, Marie Saint-Hilaire, Linda Tickle-Degnen

**Affiliations:** 1 Department of Occupational Therapy, Tufts University, 574 Boston Ave, Medford, MA 02155, USA; 2 The Gerald J. and Dorothy H Friedman School of Nutrition Science and Policy, Tufts University, 150 Harrison Ave, Boston, MA 02111, USA; 3 Department of Neurology, Parkinson’s Disease and Movement Disorders Center, Boston University Medical Center, 725 Albany St, Boston, MA 02118, USA

**Keywords:** Health Psychology, Social Psychology, Non-verbal Communication, Occupational Therapy, Parkinson’s disease, facial expression, emotion, smiling, data visualization, case series

## Abstract

People with Parkinson’s disease (PD) often experience a decrease in their facial expressivity, but little is known about how the coordinated movements across regions of the face are impaired in PD. The face has neurologically independent regions that coordinate to articulate distinct social meanings that others perceive as gestalt expressions, and so understanding how different regions of the face are affected is important. Using the Facial Action Coding System, this study comprehensively measured spontaneous facial expression across 600 frames for a multiple case study of people with PD who were rated as having varying degrees of facial expression deficits, and created correlation matrices for frequency and intensity of produced muscle activations across different areas of the face. Data visualization techniques were used to create temporal and correlational mappings of muscle action in the face at different degrees of facial expressivity. Results showed that as severity of facial expression deficit increased, there was a decrease in number, duration, intensity, and coactivation of facial muscle action. This understanding of how regions of the parkinsonian face move independently and in conjunction with other regions will provide a new focus for future research aiming to model how facial expression in PD relates to disease progression, stigma, and quality of life.

## 1. Introduction

Expression of emotion is important in maintaining social relationships that prevent loneliness and foster social support across the lifespan. Older adults with Parkinson’s disease (PD) often have diminished facial expressivity, called facial masking or hypomimia, due to impaired capacity to activate motor action in the face (Bologna et al., 2013; Bowers et al., 2006). The more severe the masking, and regardless of their actual emotional and social characteristics, the more peers and clinicians perceive people with facial masking as being depressed, apathetic, anxious, bored, and less sociable (Hemmesch, 2014; Hemmesch, Tickle-Degnen, & Zebrowitz, 2009; Pentland, Pitcairn, Gray, & Riddle, 1987; Tickle-Degnen, Zebrowitz, & Ma, 2011).

People, including clinicians, typically form impressions of the face as gestalt emotional expressions, without explicitly recognizing the underlying relationships between the different regions of the face. This is reflected in the current clinical measure of facial masking (Item 3.2 of the Movement Disorder Society’s Unified Parkinson’s Disease Rating Scale; MDS-UPDRS) which uses general impressions to classify people with PD as having none, slight, minimal, moderate, or severe facial masking (Goetz et al., 2008). These gestalt impressions are useful heuristics in making impressions of the face (e.g. Calder, Young, Keane, & Dean, 2000), but it is often activation of muscle movements in conjunction with movements in other regions of the face that produce subtle distinctions between the perceptions of emotional expressions. For example, a smile in the lower face is seen as more genuine when it is accompanied by raised cheeks in the upper half of the face (Gunnery & Ruben, 2016), and an open mouth with raised eyebrows is often perceived as surprised, while an open mouth with a furrowed brow is more often perceived as anger (Knapp, Hall, & Horgan, 2013). Since different regions of the face are motorically and neurally independent, in order to assess how facial masking influences coordinated movement across the face, facial muscle activation needs to be measured with specificity to each individual movement (Rinn, 1984).

The Facial Action Coding System (FACS) is a social psychological tool used to measure the presence, intensity, and temporal characteristics of 44 muscle movements, called action units (AUs), in the face. Previous use of FACS in people with PD has primarily investigated differences in spontaneous and deliberate facial expression and investigated use of the Duchenne smile among people with PD (Pitcairn, Clemie, Gray, & Pentland, 1990; Smith, Smith, & Ellgring, 1996). The Duchenne smile involves activation of the cheek raiser muscle (AU 6) that creates the appearance of crows’ feet around the eyes in addition to the mouth smile (AU 12) (Frank, Ekman, & Friesen, 1993) and is often perceived as showing more happiness than smiles without the cheek raiser activation (Gunnery & Ruben, 2016). Using FACS to measure smiling, a sample of four people with PD were found to produce fewer spontaneous Duchenne smiles than healthy matched controls (Pitcairn et al., 1990), however, in a sample of six people with PD some people with PD who did not produce spontaneous Duchenne smiles were able to produce a Duchenne smile when asked to do so deliberately (Smith et al., 1996). The Duchenne smile is arguably the most potent means of communicating positive emotion, and loss of the ability to produce Duchenne smiles could be quite stigmatizing.

Little is known about how facial masking is related to a person with PD’s experience of stigma and social well-being or other motor and non-motor symptoms in PD. In order to begin to understand these questions, a complete understanding of how PD affects all areas of the parkinsonian face and how different areas of the parkinsonian face are related is needed. Wu et al. (2014) began this work by creating mappings including presence and temporal aspects of 11 action units coded from 30 s videos of people with PD watching disgusting and neutral videos, but these were of induced emotional responses rather than spontaneous facial expression and did not include intensity of the movement or the relationship between activations of action units across the face.

Systematically mapping facial expression from a spontaneous narrative increases ecological validity and allows for the generalization of the measurement to the person’s actual social life. This study provides an innovative template for measuring patterns in presence, intensity, and temporal characteristics across regions of the face that are fundamental to understanding how facial masking affects social outcomes in people with PD. This type of mapping has never been done at this level of precision and will allow researchers to identify how facial masking relates to the experience of stigma and quality of life in PD as well as identify areas of the face where facial masking interventions could be helpful. This study will also provide a way to map the pathophysiological function of the face and connect changes in facial expression to other motor issues in PD such as vocal difficulties, respiratory control, gait, and postural stability.

The objectives of the current study were to use a multiple case study repeated measures design to (a) describe the facial expression of men and women with PD with differing degrees of facial masking, and (b) generate hypotheses for the ways in which a comprehensive mapping of the face in PD can be used to model social outcomes in people with PD.

## 2. Method

### 2.1. Study design

We utilized a descriptive observational multiple case study design with repeated measurement across 600 frames. This was preliminary analysis of data from the baseline assessment of the ongoing longitudinal study, Social Self-Management of Parkinson’s Disease (SocM-PD, Tickle-Degnen et al., 2014). All procedures were approved by the Institutional Review Boards where recruitment and data collection took place, and all participants gave informed consent.

### 2.2. Participants

Four men and four women were purposively selected from a sample of 55 people with idiopathic Parkinson’s disease. Participants were chosen who scored similarly for disease severity (Hoehn & Yahr stage), cognitive status, depression, and were within two standard deviations of the mean for age. Participants were allowed to vary on degree of facial masking as measured by a trained clinician’s ratings and trained behavioral coders. All participants were on anti-Parkinson medication and all were in an on state during participation. There was one participant (D) who was dyskinetic during participation. See Table 1 for a description of the participants and the overall means for the sample from which participants were selected.

### 2.3. Measures

Participants were administered the Geriatric Depression Scale (GDS; Yesavage & Sheikh, 1986) as a measure of depression. The GDS (short form) is a 15-item measure of depression with a dichotomous yes/no response format. A score of 5 or greater is indicative of clinical depression. Cognitive status was measured with the Montreal Cognitive Assessment (MoCA; Nasreddine et al., 2005), which results in a total score of correct responses with possible scores ranging from 0–30. A score of 26 is typically the cutoff for citing no cognitive impairment.

The MDS-UPDRS (Goetz et al., 2008) was also administered and the score for the facial masking item and Hoehn and Yahr stage from the scale were used. The MDS-UPDRS facial masking item measures facial masking using a 5-point categorical scale from 0 (no difficulty) to 4 (severe difficulty). The measure is completed in real time while the participant is talking and at rest, and focuses on the frequency and intensity of blinking, smiling, and mouth closure during speech in addition to rating the overall facial expressivity.

Disease severity was measured as part of the MDS-UPDRS using the Hoehn and Yahr stages. These stages are 0—asymptomatic, 1—unilateral involvement only, 2—bilateral involvement without impairment of balance, 3—mild to moderate involvement with some postural instability, 4—severe disability, still able to walk stand unassisted; 5—wheelchair bound or bedridden unless assisted.

### 2.4. Facial Action Coding System

The FACS is used to measure the presence, intensity, and temporal characteristics such as onset, offset, and apex of 44 individual visible movements called action units in the face (Ekman, Friesen, & Hager, 2002). If an action unit is coded as present, its intensity is then coded on a five-point scale from A (trace) to E (extreme). These categorical intensities can then be converted into a quantitative variable ranging from 1 to 5. Presence, intensity, and temporal aspects of facial muscle movement are measured separately which allows for a participant to vary on the three different characteristics. The FACS is completed using videotapes of participants and typically assesses facial expression frame by frame to capture very small changes in facial muscle movements. See Table 2 for a list and description of the 18 action units that were observed in the current sample. Due to the difficulty with coding for open mouth and jaw movements during speech, these action units were not included as part of the current analysis and will be addressed in future research.

### 2.5. Procedure

Participants were videotaped while telling an interviewer about a recent activity they participated in that was particularly enjoyable (Tickle-Degnen et al., 2014). This interview question has been found to reliably elicit spontaneous emotional responses in participants (Takahashi, Tickle-Degnen, Coster, & Latham, 2010). Each participant’s complete narrative was edited into 20-s clips and a group of 4–5 trained research assistants rated each 20-s clip for overall expressivity on a Likert scale from 1 (not at all expressive) to 5 (highly expressive) using methodology outlined in the Interpersonal Communication Rating Protocol (ICRP; Tickle-Degnen, 2010). Trained coders’ ratings were averaged for each clip and the 20-s clip that was rated as most expressive was selected for coding. The most expressive clip was selected to ensure that the measured expressiveness in each clip was as representative of the participant’s capacity to produce spontaneous facial expression as possible. See Table 1 for the expressivity score for each participant. These expressivity ratings were used to order participants from most to least expressive because they provided a measure of expressivity from a social situation, were more reliable than the MDS-UPDRS due to the use of multiple coders, and they were completed on the same video clips that would subsequently be rated using FACS.

A certified FACS coder (SDG) coded each 20-s clip frame by frame in Adobe Premiere Pro CC for presence and intensity of 44 action units. This resulted in 600 measurements for each participant for each action unit present and was a large enough sample to be able to generalize to each participant’s typical spontaneous expressivity. The one participant (D) with dyskinesia was coded as if all facial muscle activations were typical if the activations were not involved with speech or swallowing. While the FACS coder knew the clips were taken from enjoyable activity narratives, all coding was done without sound, and the coder was blind to MDS-UPDRS facial masking scores and expressivity scores.

### 2.6. FACS reliability

A second certified coder coded two of the eight participants to assess reliability of the coding. Reliability was determined using intraclass correlations for intensity and kappa statistics for presence of the AU (Dumer et al., 2014; Ekman et al., 2002). Both the primary and secondary coder coded the presence of 11 AUs and achieved 100% correct rejections on 32 AUs. There was a single action unit (AU 5) that the secondary coder coded at a minimal intensity for 3 frames (.10 s), and the primary coder coded as completely absent. Since this discrepancy was only for 3 of a possible 600 frames, AU 5 was excluded from the reliability analysis.

Acceptable reliability was reached for all AUs coded as present by both coders except for AU 14 (dimpler). To account for differences between coders, both coders reached consensus for AU 14 across all eight participants. Including agreed upon coding of AU 14, the two coders showed good reliability with an average kappa = .74 and average intraclass correlation of .84.

### 2.7. Data analysis

#### 2.7.1. Presence and intensity of muscle action

Intensity matrices were created for each participant with a row for every action unit and a column for every frame (0–600). For each action unit, every frame was given a value indicating the intensity at which the action unit was activated during the given frame. If the action unit was not activated during that frame it was given a value of 0. If the action unit was activated it was given a value from 1 (minimal activation) to 5 (extreme activation).

#### 2.7.2. Correlations between action units within the face

For each participant, a series of Spearman Rho correlations were calculated between the intensity ratings described above for each action unit present across the 600 frames. This created a correlation coefficient for the relationship between the intensities of each action unit that a participant produced during the 20-s clip and showed how the intensity of each muscle movement covaried with the intensity of other action units across time. Each correlation had a sample size of 600. Because of the large sample size all correlation coefficients greater than *r* = .08 were significant and so we present correlations as effect sizes and indicate the direction and strength of the relationship rather than interpreting the *p* value associated with the correlations.

#### 2.7.3. Visualization of muscle action

**Figure 1:**
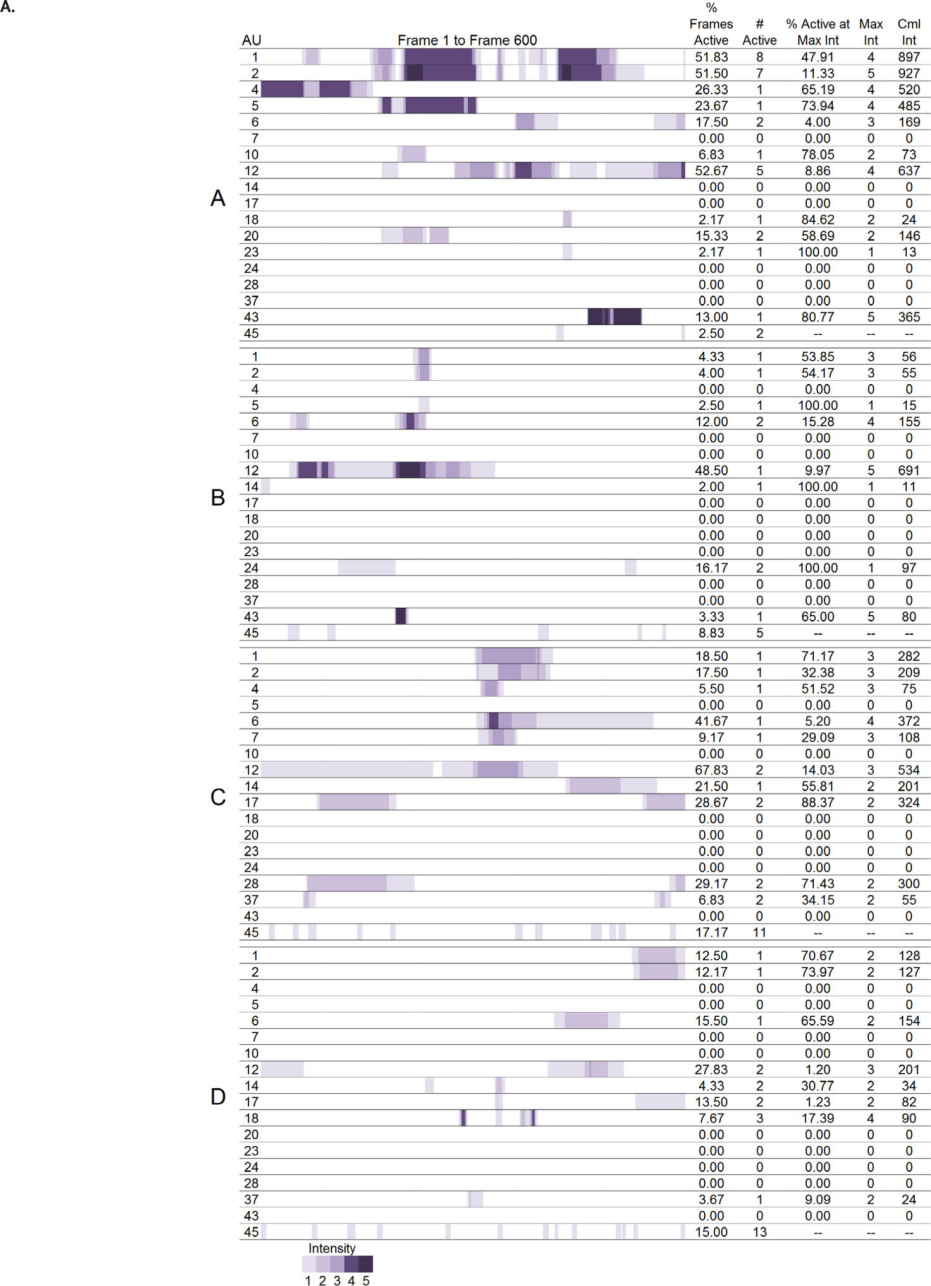

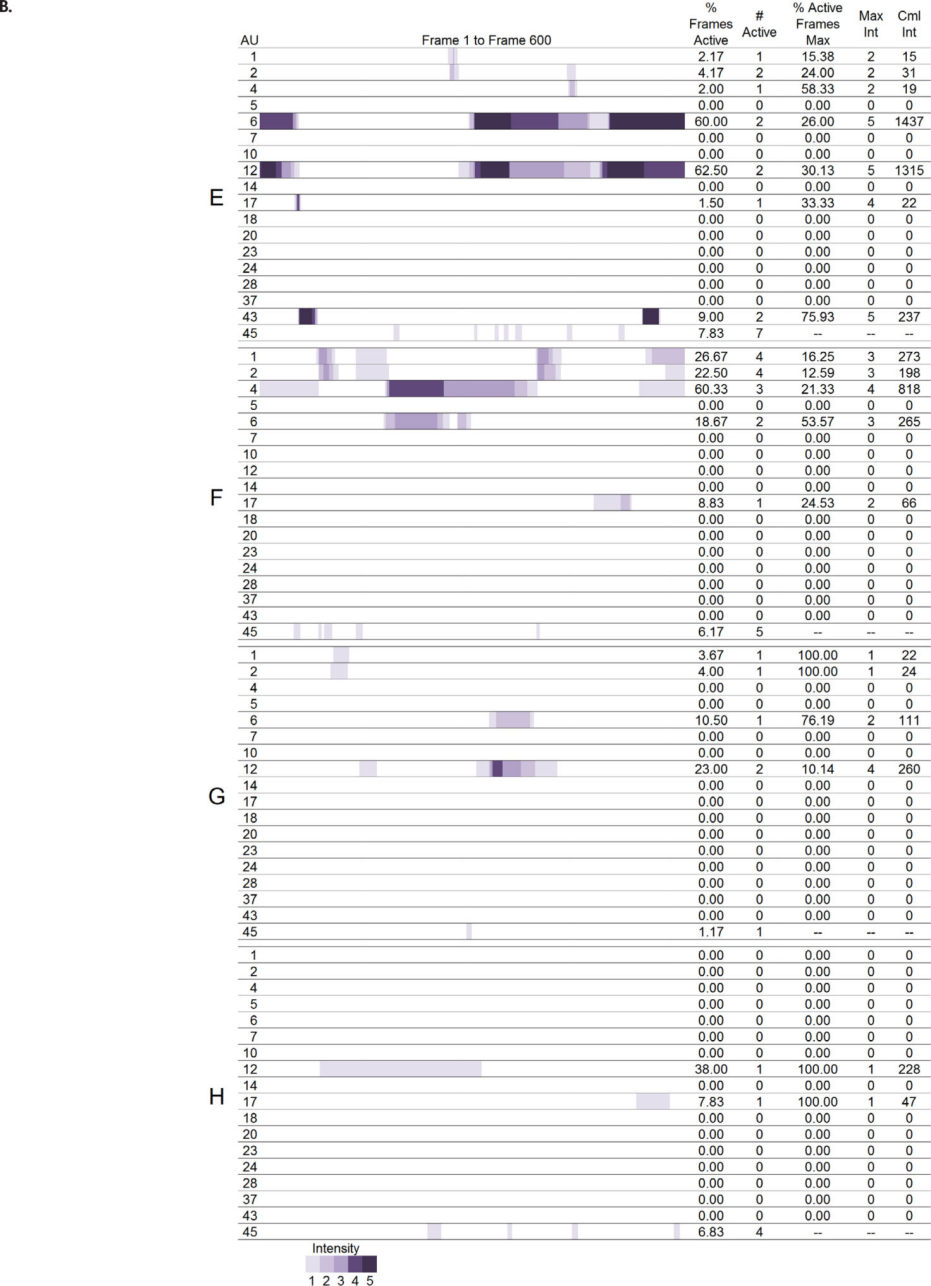
A. Visualization of activation and intensity of AUs across 600 frames and summary statistics for women. B. Visualization of activation and intensity of AUs across 600 frames and summary statistics for men.

Maps showing the time course and changes in intensity of each action units were developed to visualize the digitized facial muscle activation for each participant. The digitization figures show when and at what intensity each action unit was activated across the 600 frames. The space is colored purple when an action unit was active and the darker the shade of purple the more intense the action was. Moving left to right across the figures are the frames from 0–600. Multiple bands of purple separated by white areas indicate that the action unit was activated more than once during the 600 frames. See Figures 1A and 1B.

**Figure 2:**
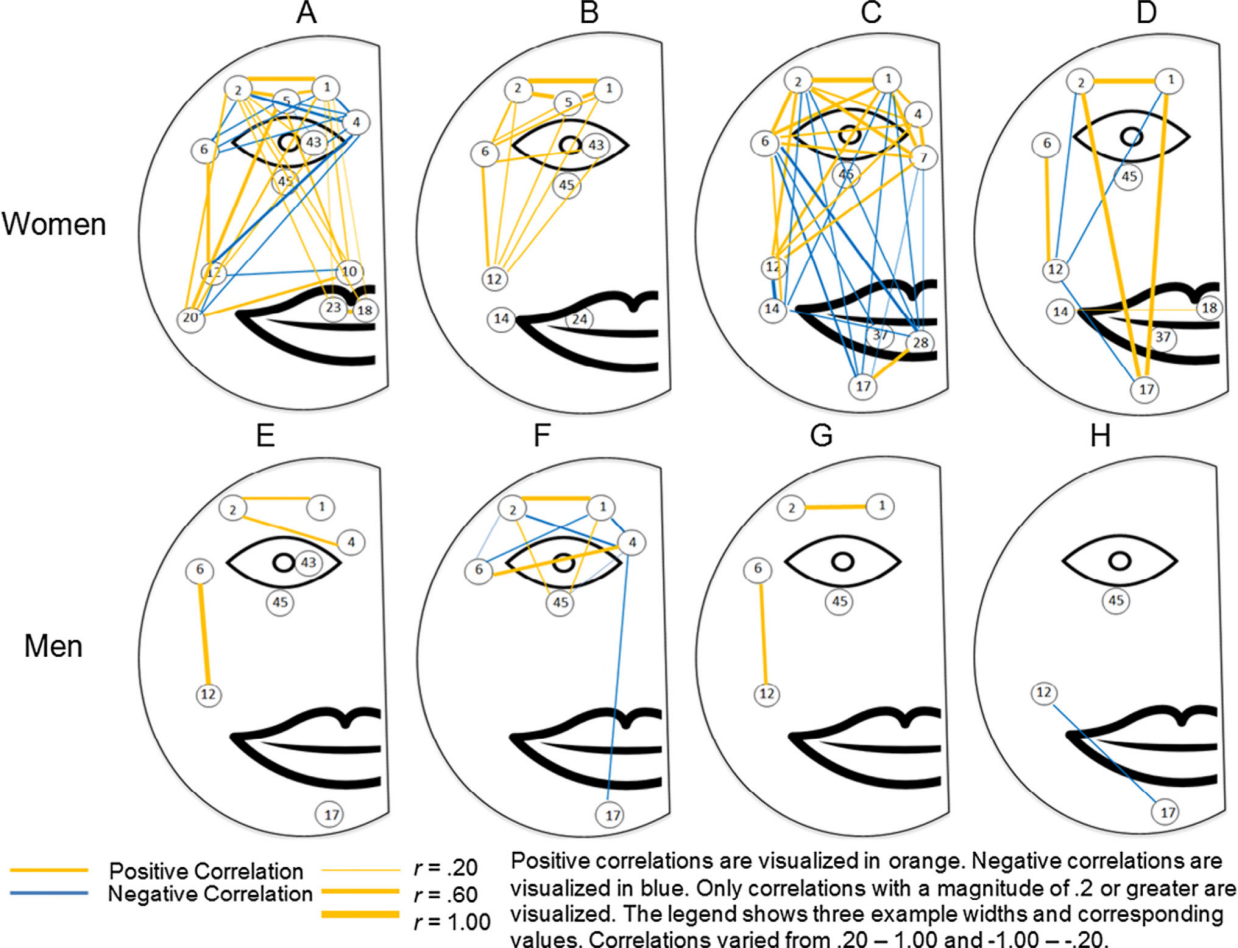
Intensity correlations between AUs for women and men.

Maps of the spatial structure and intercorrelations of activation in the face were created for each participant showing their main areas of activation and coactivation. Correlations between activated action units with a magnitude greater than .20 or −.20 were visualized for each participant. We chose this cut-off to be conservative as a correlation of *r* = .20 with 598 degrees of freedom corresponds to *p* < .00001. Positive correlations were visualized in orange and negative correlations were visualized in blue. The width of the line indicates the magnitude of each relationship. See Figure 2 for visualization of correlations across the face.

#### 2.7.4. Summary metrics

Descriptive statistics that summarized the presence, intensity, and duration of each action unit were created. These included (1) the percentage of total frames that an action unit was active, (2) the number of discrete times each unit was activated, (3) the percentage of active frames that the action unit was active at its maximum intensity, (4) the maximum intensity at which the action unit was produced, and (5) the cumulative intensity of the action unit’s activation across the 600 frames (the sum of the intensity values for each action unit across the 600 frames). Since blinks are always coded at an intensity of one, the only summary statistics reported for that action unit are the percent of frames active and the number of times the person blinked. Summary statistics were then correlated across action units (sample sizes ranged from 3–12 based on the number of action units each participant produced) for each participant to show the relationships between the different summary statistics. The mean correlations across the eight participants were calculated. For mean correlations, all correlation coefficients were Fisher Z transformed to normalize the distribution, averaged, and converted back to *r* for ease of presentation.

## 3. Results

### 3.1. Individual results

Due to the large number of results, we will present individual results for the most expressive woman, A, and the most expressive man, E, for the action units involved in the Duchenne smile: the cheek raiser (AU 6) and lip corner puller (AU 12) as a case, and present all other results in Figure 1A and 1B and in Table 3. Figure 1A and 1B displays the digitization of muscle activation and the summary statistics for women and men, respectively. Table 3 displays the action unit that received the greatest value and the corresponding value for each of the summary statistics. It should be noted that for the action unit present at maximum intensity for the greatest percentage of active frames, the action unit had to be active at an intensity of 2 or greater as action units active at a maximum intensity of 1 would give a value of 100% for this column.

Participant A activated AU 6 in 17.50% of the 600 frames. She activated the action unit two distinct times at a maximum intensity of 3. AU 6 was active at an intensity of 3 for 4.00% of the frames and had a cumulative intensity of 169. Participant A activated AU 12 in 52.67% of the 600 frames. She activated the action unit five distinct times at a maximum intensity of 4. AU 12 was active at an intensity of 4 for 8.86% of the frames and had a cumulative intensity of 637.

Participant E activated AU 6 in 60.00% of the 600 frames. He activated the action unit two distinct times at a maximum intensity of 5. AU 6 was active at an intensity of 5 for 26.00% of the frames and had a cumulative intensity of 1,437. Participant E activated AU 12 in 62.50% of the 600 frames. He activated the action unit two distinct times at a maximum intensity of 5. AU 12 was active at an intensity of 5 for 30.13% of the frames and had a cumulative intensity of 1,315.

Both the most expressive woman and the most expressive man produced 2 Duchenne smiles during their most expressive 20 s clip. Overall, Participant A smiled (AU 12) more times, and Participant E smiled more intensely and at a higher intensity for a greater proportion of frames.

### 3.2. Individual correlational results

Figure 2 shows the Spearman Rho correlations of the intensity of the action unit activation across the 600 frames. Table 4 presents the correlation coefficients for the correlations presented in Figure 2. Using Participants A and E and AUs 6 and 12 as a case once again, the correlation between intensities of AU 6 and AU 12 for Participant A was *r*(598) = .65, *p* < .001 and for Participant E was *r*(598) = .95, *p* < .001. There was a strong and positive correlation between the cheek raiser (AU 6) and lip corner puller (AU 12) for both Participant A and Participant E. The correlation for Participant E approached 1.00. This strong correlation can be seen by looking at how the coloring for the AU 6 and AU 12 row in Figure 1B covary together although the intensity for AU 6 is consistently higher across the 600 frames than the intensity of 12. See Figure 2 and Table 4 for the remainder of the correlations between action units for each participant.

### 3.3. Common patterns across all participants

#### 3.3.1. Patterns of activation

Table 5 provides the summary statistics averaged across all eight participants. Across the eight participants, the number of action units activated ranged from three (Participant H) to 12 (Participant A) with a cumulative total of 18 actions produced across the eight participants (Table 2). Across all eight participants, the lip corner puller (AU 12) was the action unit produced for the highest percentage of total frames, at the second highest average intensity, and the greatest cumulative intensity. Eye closure (AU 43) had the highest average maximum intensity because all participants who activated their eye closure closed their eyes completely. All but one participant (F) smiled (AU 12) during their narrative. The percent of active frames where action units were activated at maximum intensity was higher for action units with lower maximum intensities indicating that participants did not maintain activation of action units at higher intensities for as long as they did action units activated at lower intensities. The cheek raiser (AU 6) followed the lip corner puller with the third highest percentage of active frames, mean intensity, and second highest cumulative intensity. Of the six people who smiled (A, B, C, D, E, G, H), only Participant H did not activate his cheek raiser while smiling and thus produce a Duchenne smile. The inner (AU 1) and outer (AU 2) brow raisers were produced the greatest number of times. There were six AUs that were only produced by one participant. These were AUs 7 (lid tightener, produced by C), 10 (upper lip raiser, A), 20 (lip stretch, A) 23 (lip tightener, A), 24 (lip presser, B), and 28 (lip suck, B).

The correlations between summary statistics averaged across the eight participants (presented in Table 6) show that participants who tended to have higher percentages of active frames also tended to have a higher maximum intensity for that action unit and a higher cumulative intensity. Maximum intensity and cumulative intensity were also strongly correlated in a positive direction. The greater the number of times participants produced an action unit, the more likely they were to activate the action unit at a high intensity, but number of activations was negatively and moderately correlated with maintaining activation at the maximum intensity. The percentage of active frames where the action was produced at maximum intensity was negatively and strongly correlated with maximum intensity. This correlation further confirms that participants were more likely to maintain activation of the action unit at its maximum intensity if the maximum intensity reached was not very intense.

#### 3.3.2. Patterns in correlations between individual action units

Across all participants certain action units showed consistent correlations. The most consistently observable correlation was between the inner brow raiser (AU 1) and outer brow raiser (AU 2), which were positively correlated at a high magnitude for all participants that produced the two actions. This is not unexpected as the inner and outer brow raisers are typically activated concurrently.

The lid raiser was also strongly positively correlated with both eyebrow raisers, but this action unit was only produced by two participants (A and B). A lack of correlations between any action units and the blink (excluding Participant F) indicates that blinking was largely independent of other action in the face. There were no other clear patterns in the upper face.

Correlations between the top and bottom half of the face showed that lip corner puller (AU 12) and cheek raiser (AU 6) were positively correlated at a moderate magnitude across five of the six participants (all but C) that produced the two action units.

There were less discernible patterns in the lower face. Lip corner puller (AU 12) was frequently negatively correlated with other AUs in the bottom half of the face indicating that while participants were smiling they were not activating other muscles (i.e. AUs 10, 14, and 17).

#### 3.3.3. Gender differences

A comparison of Figures 1A, 1B, and 2 shows clear gender differences in the patterns that emerged. Women produced more action units across degrees of facial masking severity than men, but men showed more evidence of concordance in their activation. The visualizations of the women’s activation showed more random patterning, while the men were less likely to produce action units that were not concurrently produced with other action units. These visual patterns were seen further in the correlations depicted in Figure 2. Men produced mostly positive correlations indicating that individual action units were more likely produced in concurrence with other action units than were produced independently. Blinking patterns were also different in men and women.

Blinking increased as facial expressivity deficits increased in women with the least expressive woman blinking the most and the most expressive woman blinking the least. Participant A blinked twice while participant D blinked 13 times. Blinking patterns in the men were more random but showed a trend toward less expressive men blinking less than the most expressive men. Participant E blinked seven times while participant H blinked only four times.

There were no gender differences in intensities of action units produced. For both men and women, there was an increase in the intensity of the action units produced from lowest to highest degrees of facial expressivity with Participant E, the most expressive man, producing the most intense activation for the longest duration.

## 4. Discussion

Results demonstrate that these innovative methodologies are able to detect and visualize characteristics of facial expression in people with PD that can be used to distinguish between degrees of facial masking severity and also describe how men with PD differ from women with PD during naturalistic social interactions. These results indicate that this methodology is able to go beyond what is gained from the single holistic rating of facial expressivity to show the more subtle and detailed differences in facial muscle action in different areas of the face in people with PD. The characteristics that most clearly differed between participants were the number of different muscles that were activated, the intensity of the muscle activation, the duration of the activation, and the correlations between different action units activated.

The number of different muscles activated differed between genders in this sample with women contracting more muscles than men, and differed less so between degrees of facial masking severity within genders. Intensity of muscle activation was a good indicator of facial masking in both genders, but there were especially clear differences in intensity of the muscle action produced in men with the least expressive man only displaying minimal activation and the most expressive man displaying extreme activation. The percent of possible frames in which an action unit was active was a good predictor of degree of facial masking severity, as people with less masking had higher percentages of activation. Negative correlations between action units in the face, which were evidence of discordant muscle activation, or muscles moving independently of other muscles, were more characteristic of women. Overall, individuals with more facial masking tended to produce muscle movements concurrently, except Participant H, who did not show enough activation in his face to adequately test this. It is possible that this is confounded by the overall number of muscles activated and needs to be replicated in future studies.

Previous research on facial expression in PD has found that people with PD produce less spontaneous Duchenne smiles than healthy controls (Smith et al., 1996). The present study found that these participants with PD were capable of producing Duchenne smiles (AU 6 + AU 12) but the frequency and intensity decreased as facial masking increased. The most typically expressive participants, A and E, produced multiple intense Duchenne smiles that were active for longer durations than the participants with greater facial expression deficits.

A limitation of the study is that gender may be confounded with facial masking severity as all women participants were more expressive than their male counterparts with the same degree of masking. Previous research on nonverbal behavior in populations with typical facial expressions generally shows that women are more expressive than men (Kring & Gordon, 1998), but further research is needed to demonstrate the generalizability of the gender differences found in the current study.

Another limitation of the study is that some participants selected may not have expressive behavior that is characteristic of other people with PD at their degree of masking. Participant H showed very little activation in his face which led to missing data for correlations of summary statistics, while Participant D, the least expressive woman in the study, showed much more activation than Participant H, but had some dyskinesia in her face that may have influenced how her muscle action was coded. Her dyskinesias are likely what produced the intense and frequent lip pucker observed in her data, but further work using these methodologies is needed to better distinguish between dyskinetic and non-pathological movements. Replicating these procedures with larger sample sizes will allow researchers to better generalize to the population of people with PD as a whole, as well as determine the role of dyskinesia in the expression of people with PD. This multiple case study provides evidence for the feasibility and value of using these methodologies with a larger and more diverse sample of participants.

Though these findings are limited in their ability to generalize to the population of people with PD as a whole, they are novel in their ability to generalize to each participants’ overall spontaneous behavior and in how they describe how people with PD with varying degrees of expressivity deficits move their faces in the absence of comorbid depression or major cognitive impairment. These findings also show the feasibility of conducting hypothesis testing and model building with a larger sample size. Our future research will test the hypothesis that characteristics of muscle action in the face can predict experience of stigma in people with PD and impression formation of observers as well as distinguish facial masking from comorbidities that affect the face such as depression and cognitive impairment. This future research will also investigate how characteristics of the face map onto and predict other motor symptoms seen throughout the body such as bradykinesia, tremor, dyskinesia, and rigidity. The long-term goals of our future research are to tailor non-pharmaceutical interventions to patients that can increase their ability to communicate their positive emotion and decrease stigma thus improving quality of life.

## Figures and Tables

**Table 1:** Participant demographics and overall sample central tendency

Women							Men						
ID	Age	MoCA	GDS	Expr	UPDRS face	Disease severity	ID	Age	MoCA	GDS	Expr	UPDRS face	Disease severity
A	69	26	1	5.00	Slight	Bilateral (2)	E	62	24	2	4.80	Slight	Bilateral (2)
B	67	29	1	4.25	Slight	Bilateral (2)	F	63	27	3	3.50	Slight	Unilateral (1)
C	72	27	1	3.20	Mild	Bilateral (2)	G	60	26	3	2.60	Mild	Bilateral (2)
D	67	24	1	2.40	Slight	Severe (4)	H	66	26	0	1.50	Moderate	Severe (4)
Sample	63	26	3.39	3.65	Slight	Bilateral (2)	Sample	66	25	1.63	2.62	Mild	Bilateral (2)

Notes: Within genders, participants are ordered by their expressivity score. MoCA = Montreal Cognitive Assessment, GDS = Geriatric Depression Scale, Expr = trained coder rated expressivity, UPDRS face = Unified Parkinson Disease Rating Scale Facial Item Rating, Disease severity = Hoehn and Yahr disease severity rating, corresponding numerical scores are in parentheses; Statisitics in the row Sample are mean or medians for the whole sample of 55 participants from which the 8 participants were purposefully selected.

**Table 2:** Number and description of observed action unit

Action unit #	Description
1	Inner brow raise
2	Outer brow raise
4	Brow lowerer
5	Upper lid raise
6	Cheek raise
7	Lids tight
10	Upper lip raiser
12	Lip corner puller
14	Dimpler
17	Chin raiser
18	Lip pucker
20	Lip stretch
23	Lip tightener
24	Lip presser
28	Lip suck
37	Lip wipe
43	Eye closure
45	Blink

**Table 3:** Summary of activation for each participant

Case	# AUs produced	AU with largest % frames active	AU with greatest # of activations	AU with greatest % active frames at max intensity	AU with highest intensity	AU with greatest cumulative intensity	# Blinks
A	12	12 (52.67)	1(8)	18 (84.62, 2)	2, 43 (5)	2 (927)	2
B	9	12 (48.50)	6, 24 (2)	43 (65.00, 5)	12, 43 (5)	12 (691)	5
C	11	12 (67.83)	12, 17, 28, 37 (2)	17 (88.37, 2)	6 (4)	12 (534)	11
D	9	12 (27.83)	18 (3)	2 (73.97, 2)	18 (4)	12 (201)	13
E	8	12 (62.50)	2, 6, 12, 43 (2)	43 (78.93, 5)	6, 12, 43 (5)	6 (1,437)	7
F	6	4 (60.33)	1, 2 (4)	6 (53.57, 3)	4 (4)	4 (818)	5
G	5	12 (23.00)	12 (2)	6 (76.19, 2)	12 (4)	12 (260)	1
H	3	12 (38.00)	12, 17 (1)	–	12, 17 (1)	12 (228)	4

Notes: AU = Action unit. For all columns that present the AU that received the largest value for each category, the number in parentheses is the corresponding value for the AU. AU with greatest % active frames at max intensity presented the AU that was active at its maximum intensity for the greatest percentage of frames only for AUs that were active at an intensity greater than one. Participant H does not have a value for greatest % active frames at max intensity because he only produced AUs at an intensity of one.

**Table 4:** Correlations of action units across the face for each participant

Case	Upper face		Lower face		Between upper and lower	
	Action units	*r*	Action units	*r*	Action units	*r*
A	1 & 2	.88	10 & 12	−.26	1 & 10, 18, 20, 23	.21 – .43
	2 & 43	.24	10 & 20	.65	2 & 10, 18, 20, 23	.25 – .45
	4 & 1, 2, 5, 43	−.56 − −.23	12 & 20	−.29	4 & 12	−.58
	5 & 1, 2	.61			4 & 20	−.25
	5 & 43	−.21			5 & 10	.48
	6 & 1, 2, 4, 5	−.31 − −.23			5 & 20	.76
					6 & 12	.65
B	1 & 2	.96	N/A		12 & 1, 2, 5, 6	.24 – .55
	1 & 5	.77				
	2 & 5	.80				
	6 & 1, 2, 5, 43	.30 – .50				
C	1 & 2, 4, 6, 7	.53 – .97	12 & 14	−.69	1 & 14, 17, 28	−.30 − −.25
	2 & 4, 6, 7	.48 – .69	14 & 28	−.33	2 & 14, 17, 28	−.29 − −.24
	4 & 6	.40	17 & 28	.71	6 & 14	.44
	4 & 7	.76			6 & 17, 28, 37	−.52 − −.22
	6 & 7	. 53			7 & 17, 28	−.20
					12 & 1, 2, 4, 7	.43–.59
D	1 & 2	.99	12 & 17	−.24	1 & 17	.89
			14 & 18	.21	2 & 17	.90
					6 & 12	.73
					12 & 1, 2	−.23
E	1 & 2	.61	N/A		6 & 12	.94
	2 & 4	.56				
F	1 & 2	.83	N/A		4 & 17	−.34
	1 & 4	−.37				
	1 & 6	−.26				
	1 & 45	.30				
	2 & 4	−.43				
	2 & 6	−.20				
	2 & 45	.36				
	4 & 6	.66				
	4 & 45	−.20				
G	1 & 2	.87	N/A		6 & 12	.72
H	N/A		12 & 17	−.23	N/A	

Notes: In the action unit columns numbers reported refer to the specific AUs: 1 = inner brow raise, 2 = outer brow raise, 4 = brow lowerer, 5 = upper lid raiser, 6 = cheek raise, 7 = lids tight, 10 = upper lip raiser, 12 = lip corner puller, 14 = dimpler, 17 = chin raiser, 20 = lip stretch, 28 = lip suck, 45 = blink. N/A indicates there were no correlations in that region of the face. Ranges are given when action units are correlated with three or more other action units in that region of the face in the same direction. All correlations have an *n* = 600 and *p* < .00001.

**Table 5:** Summary statistics averaged across all eight participants

AU	*N*	% Frames active	# Active	% Active at max int	Max int	Cml int
1. Inner brow raise	7	17.10	2.43	53.60	2.57	239.00
2. Outer brow raise	7	16.55	2.43	44.06	2.71	224.43
4. brow lowerer	4	23.54	1.50	49.09	3.25	358.00
5. Upper lid raise	2	13.08	1.00	86.97	2.50	250.00
6. Cheek raise	6	25.12	1.57	35.12	3.29	380.43
7. Lids tight	1	9.17	1.00	29.09	3.00	108.00
10. Upper lip raiser	1	6.83	1.00	78.05	2.00	73.00
12. Lip corner puller	7	45.76	2.14	24.90	3.57	552.29
14. Dimpler	3	9.28	1.33	62.19	1.67	82.00
17. Chin raiser	5	12.07	1.40	49.49	2.20	108.20
18. Lip pucker	2	4.92	2.00	51.00	3.00	57.00
20. Lip stretch	1	15.33	2.00	58.69	2.00	146.00
23. Lip tightener	1	2.17	1.00	100.00	1.00	13.00
24. Lip presser	1	16.17	2.00	100.00	1.00	97.00
28. Lip suck	1	29.17	2.00	71.43	2.00	300.00
37. Lip wipe	2	5.25	1.50	21.62	2.00	39.50
43. Eye closure	3	8.44	1.33	73.90	5.00	227.33
45. Blink	8	8.19	6.00	–	–	–

Notes: *N* = The number of participants that produced the AU, % Frames active = Percent of the total 600 frames where the AU was active, # Active = The number of times a participants activated the AU during the 600 frames, % Active at max int = the percentage of active frames where the AU was activated at its maximum intensity, Max int = The maximum intensity at which the AU was activated, Cml int = The cumulative intensity or total of all intensity codes across the 600 frames.

**Table 6:** Correlations among summary statistics averaged across all 8 participants

	1.	2.	3.	4.
1. % Frames active				
2. Number of activations	.29			
3. % Of active Frames at max intensity	−.63	−.48		
4. Maximum intensity	.76	.50	−.65	
5. Cumulative intensity	.98	.53	−.51	.79

Notes: % Frames active = Percent of the total 600 frames where the AU was active, % of Active at max intensity = the percentage of active frames where the AU was activated at its maximum intensity.

## PUBLIC INTEREST STATEMENT

Older adults with Parkinson’s disease are often misperceived by others as apathetic, depressed, or cognitively impaired due to having a masked, rigid facial expression. Little is known about how the facial mask affects muscle movement in the parkinsonian face and thus little can be done to help older adults with Parkinson’s disease communicate happiness more accurately. The current work mapped the effects of facial masking on spontaneous facial expression in older adults with Parkinson’s disease. We found that as severity of facial expression deficit increased, mappings showed a detectable decrease in the number, duration, intensity, and coactivation of muscle movement across the face. This understanding of how regions of the parkinsonian face move will provide a new focus for future research aiming to model how facial expression in PD relates to disease progression, stigma, and quality of life.
